# Supplementary figures and images for: Citrobacter rodentium infection activates colonic lamina propria group 2 innate lymphoid cells
Source: PLoS Pathog. 2025 Jul 1;21(7):e1013276. doi: 10.1371/journal.ppat.1013276 (PMC12237274; doi:10.1371/journal.ppat.1013276)

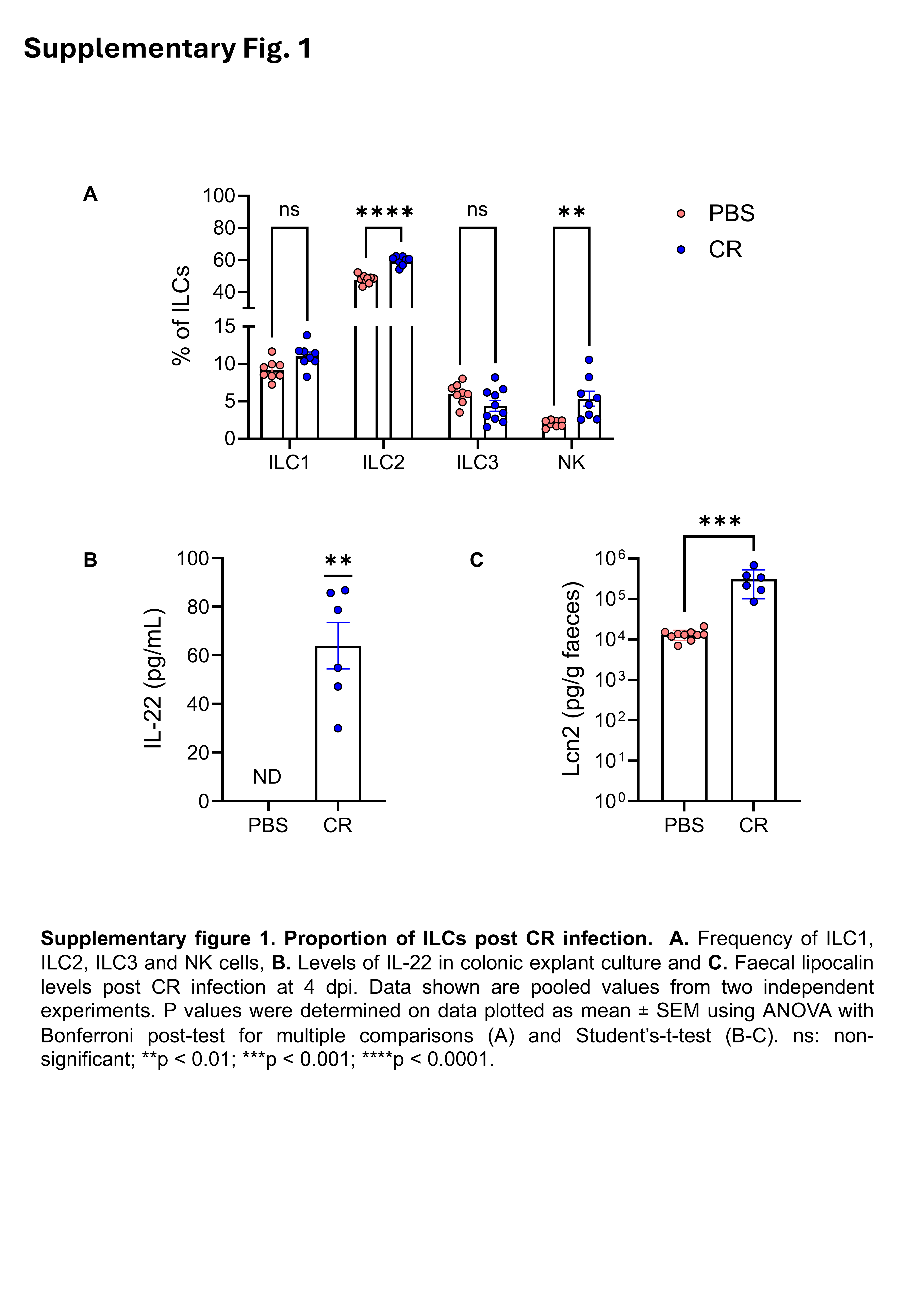

Supplement: S1 Fig — A. Frequency of ILC1, ILC2, ILC3 and NK cells, B. Levels of IL-22 in colonic explant culture and C. Faecal lipocalin levels post CR infection at 4 dpi. Data shown are pooled values from two independent experiments. P values were determined on data plotted as mean ± SEM using ANOVA with Bonferroni post-test for multiple comparisons (A) and Student’s-t-test (B-C). ns: nonsignificant; **p < 0.01; ***p < 0.001; ****p < 0.0001. (TIF) [file ppat.1013276.s001.tif]

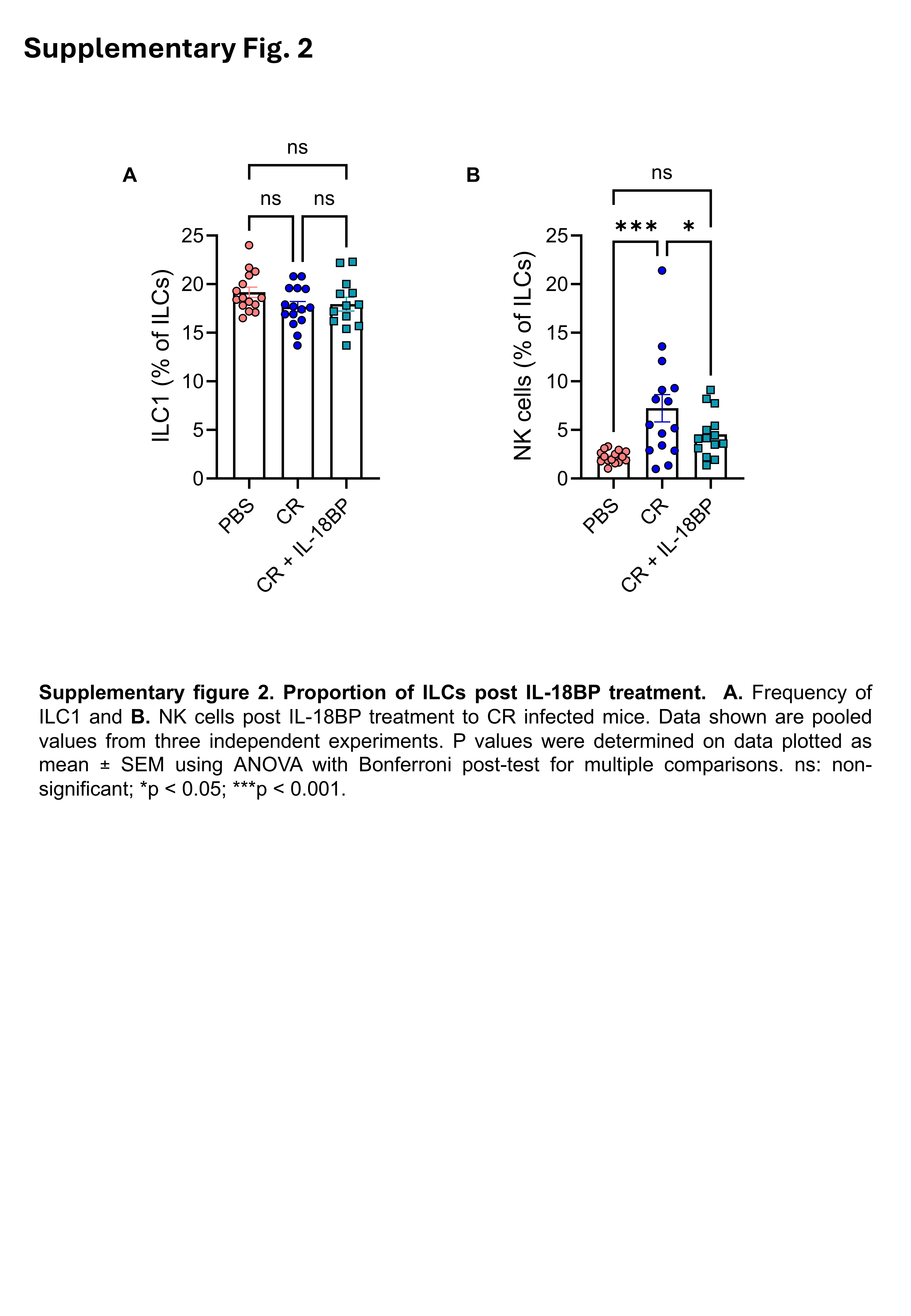

Supplement: S2 Fig — A. Frequency of ILC1 and B. NK cells post IL-18 BP treatment to CR infected mice. Data shown are pooled values from three independent experiments. P values were determined on data plotted as mean ± SEM using ANOVA with Bonferroni post-test for multiple comparisons. ns: nonsignificant; *p < 0.05; ***p < 0.001. (TIF) [file ppat.1013276.s002.tif]

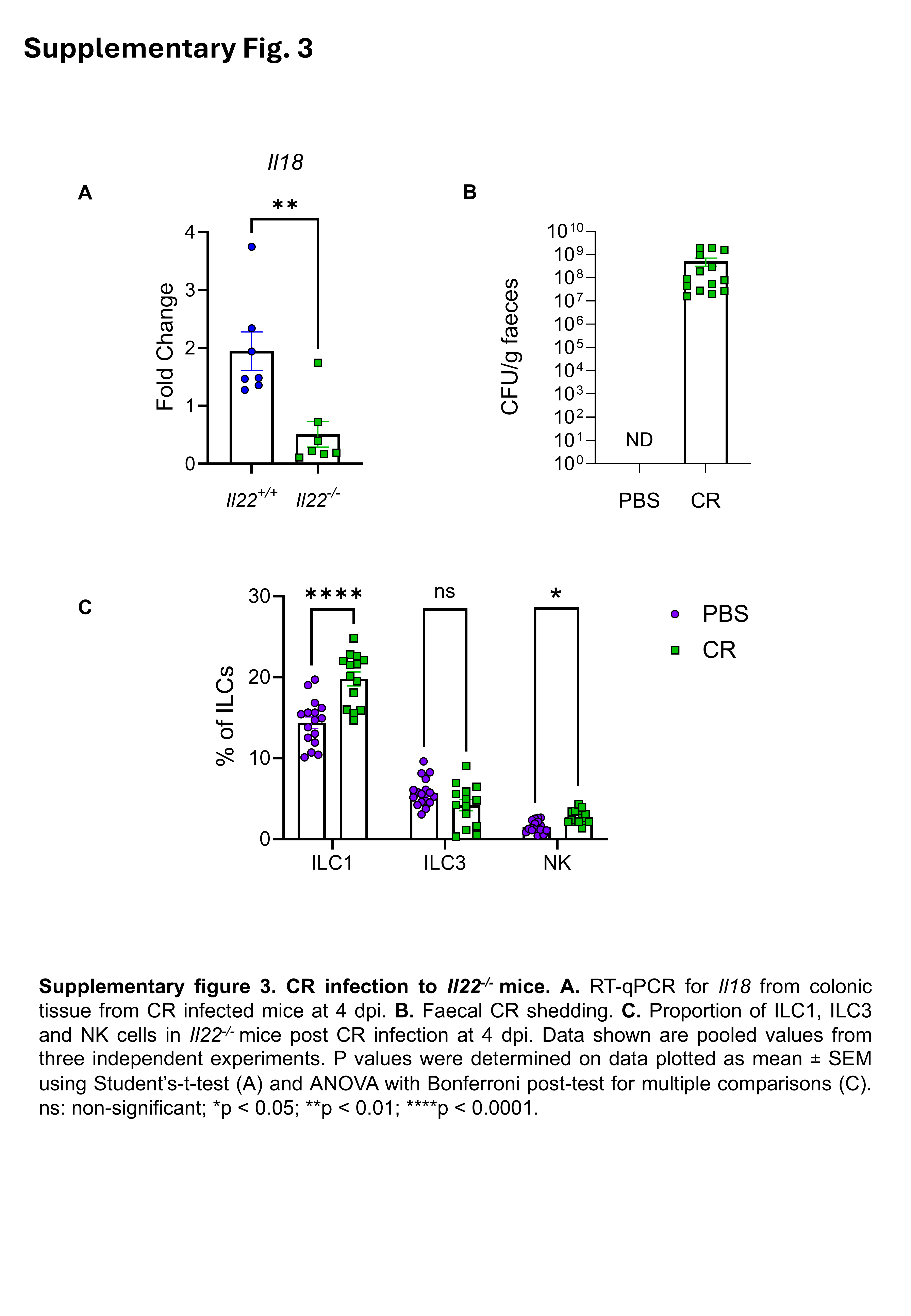

Supplement: S3 Fig — A. RT-qPCR for Il18 from colonic tissue from CR infected mice at 4 dpi. B. Faecal CR shedding. C. Proportion of ILC1, ILC3 and NK cells in Il22-/- mice post CR infection at 4 dpi. Data shown are pooled values from three independent experiments. P values were determined on data plotted as mean ± SEM using Student’s-t-test (A) and ANOVA with Bonferroni post-test for multiple comparisons (C). ns: non-significant; *p < 0.05; **p < 0.01; ****p < 0.00. (TIF) [file ppat.1013276.s003.tif]

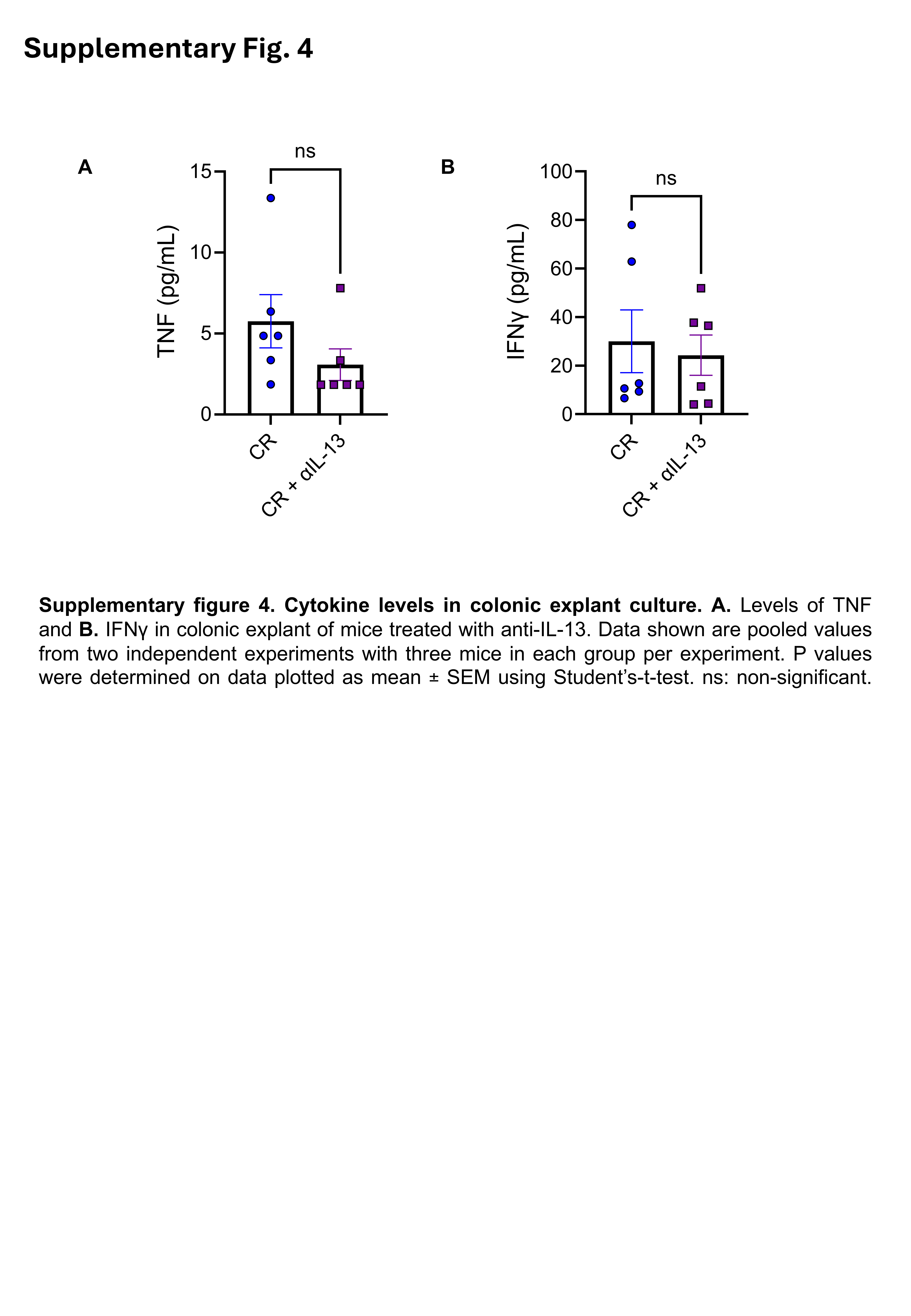

Supplement: S4 Fig — A. Levels of TNF and B. IFNγ in colonic explant of mice treated with anti-IL-13. Data shown are pooled values from two independent experiments with three mice in each group per experiment. P values were determined on data plotted as mean ± SEM using Student’s-t-test. ns: non-significant. (TIF) [file ppat.1013276.s004.tif]

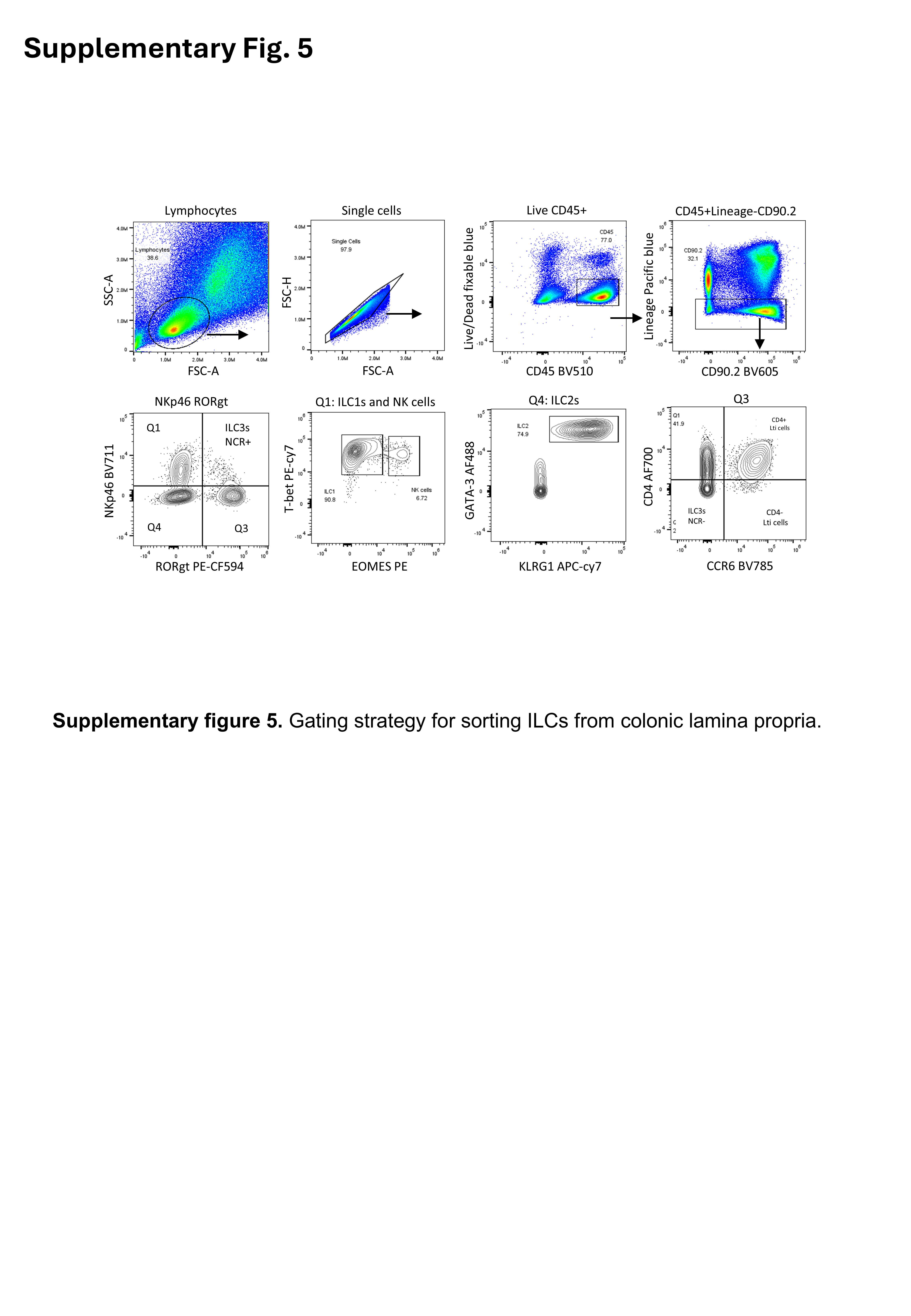

Supplement: S5 Fig — (TIF) [file ppat.1013276.s005.tif]
